# Unilateral vulval swelling in a cyclist: A case report

**DOI:** 10.1016/j.jdcr.2024.06.035

**Published:** 2024-07-23

**Authors:** Farrah Aljuhani, Jean-Philippe Arnault, Guillame Chaby

**Affiliations:** CHU Amiens, Amiens, France

**Keywords:** bicycling, sexual health, sport, swelling, vulva

## Introduction

Bicycle riding has been associated with a number of health risks in both males and females including orthopedic injuries, pelvic neurovascular compromise, and complications related to urogenital system, causing genital numbness, erectile dysfunction, priapism, infertility, and hematuria.^1^, ^2^, ^3^

Moreover, many chronic injuries related to intensive cycling in female individuals specifically have been recognized, most commonly urogenital complaints such as friction dermatitis, folliculitis, ecchymosis, hematoma, hematuria, clitoral microcalcification, difficulty in achieving orgasm, perineal numbness, iliac vein syndrome, and pudendal neuralgia.^4^^,^^5^ A new clinical problem has been observed: bicyclist’s vulva. It is characterized by painless vulvar swelling that is usually unilateral.^3^ This condition has recently been described and is still under-recognized by dermatologists and gynecologists because of its nonspecific symptoms and rarity in the literature. To date, fewer than 25 cases have been reported. In this article, we report a case of an adult female cyclist with bicyclist’s vulva that was misdiagnosed for 1 year.

## Case report

A 47-year-old female individual first visited our outpatient clinic in March 2023 with bilateral vulvar edema since 1 year. She was initially diagnosed with genital herpes and treated with valaciclovir for 1 year without improvement. Her medical history included Lynch syndrome complicated with endometrial cancer, which was treated with total hysterectomy, bilateral adnexectomy, and brachytherapy. She had been a competitive, long-distance cyclist for 25 years. Her vulvar swelling on the left side was intermittent and aggravated by heavy training. There was no family history of lymphedema or other risk factors for vulvar lymphedema.

**Fig 1:**
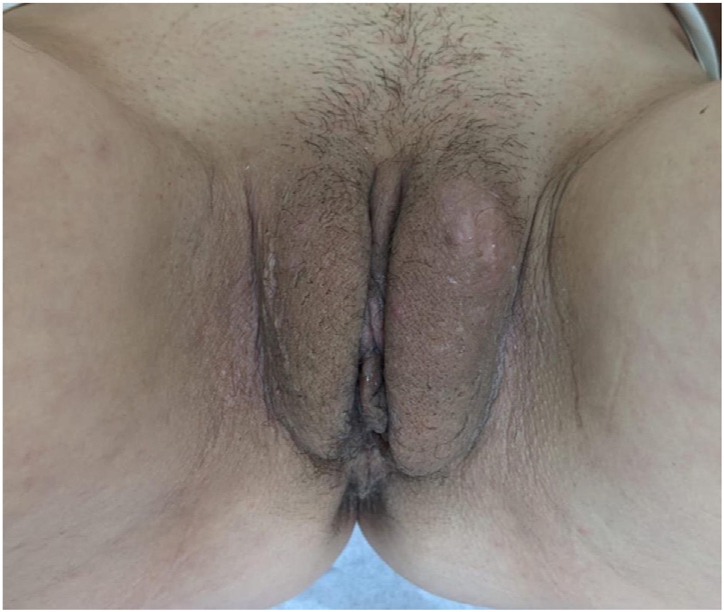
Physical examination showing bilateral symmetrical vulvar swelling with small punctures and folliculitis.

Physical examination revealed bilateral symmetrical vulvar swelling with small punctures and folliculitis (Fig 1). There were no fistulas, signs of hidradenitis suppurativa, Crohn’s disease, or lichen sclerosis. Further examination revealed no lymphadenopathy. Ultrasound examination revealed no underlying lymphatic malformations. The edema was limited to the labia majora. No involvement of the labia minora or clitoris was observed.

After reviewing the literature and excluding other diseases, a diagnosis of bicyclist’s vulva was concluded.

## Discussion

Bicyclist’s vulva is defined as a unilateral or bilateral chronic swelling of the labium majus seen in female high-level cycling competitors and is correlated with more intense and longer training.^4^^,^^6^ It was first reported by Humphries in 2002.^7^ The swelling was not consistent with lymphedema but was associated with folliculitis of the overlying skin.

Since 2002, 18 cases of bicyclist’s vulva have been reported.^5^, ^6^, ^7^ This emerging clinical problem remains under-recognized by dermatologists and gynecologists because of the nonspecific symptoms and few reports in the literature. This is consistent with the case of our patient who was misdiagnosed with a vulvar herpetic infection for 1 year.

Our patient was a middle-aged woman, consistent with that observed in previous studies in that the condition most commonly occurs in adulthood.^5^, ^6^, ^7^

Vulvar lymphedema may be caused by a combination of chronic inflammation in the vulvoperineal area, which is very common in competitive cyclists, and microtrauma to the lymphatic vessels. Another factor is the chronic pressure on the inguinal lymphatic vessels due to the curved posture of the cyclists, leading to obstruction of the lymphatic drainage; thus, localized lymphedema has been described.^4^^,^^7^

Treatment should primarily include adjustment of the bicycle to reduce the risk of perineal and vulvar microtrauma, along with the application of ice packs immediately after cycling. Additionally, elevating the legs after cycling can improve vulvar edema by improving lymphatic drainage.

## Conflicts of interest

None disclosed.
